# Migration Thresholds Govern Allele Collapse Under Repeated Gene Flooding

**DOI:** 10.1002/ece3.73740

**Published:** 2026-05-27

**Authors:** Andrew A. Davinack

**Affiliations:** ^1^ Department of Biological, Chemical, and Environmental Sciences Wheaton College Massachusetts Norton Massachusetts USA

**Keywords:** biocontrol, bioinvasion, collapse, evolutionary, simulation, swamping

## Abstract

A recent study proposed “gene flooding” as a strategy for controlling invasive populations through repeated introduction of inbred individuals. Their simulations demonstrate that sustained demographic influx can drive collapse of resident alleles under certain conditions. However, the broader dynamical structure governing when such collapse occurs remains unclear. Here, forward‐time population genetic simulations were used to examine allele replacement under repeated asymmetric migration in a density‐regulated population. Systematic variation of migration intensity revealed that allele replacement does not proceed gradually as migration increases. Instead, the system exhibits a sharp transition separating regimes of resident allele persistence from regimes of rapid allele loss. This threshold behavior remains robust across variation in mating structure and migrant reproductive disadvantage, indicating that demographic influx itself plays a dominant role in governing evolutionary outcomes. These results clarify the conditions under which migration‐driven allele replacement may occur for gene flooding to be useful and highlight the potential for nonlinear tipping points in population genetic systems experiencing sustained demographic introduction.

## Graphical abstract

A recent study proposed gene flooding as a strategy for replacing resident alleles through repeated demographic introduction of inbred individuals. Here we show that the population genetic dynamics underlying this process are governed by a sharp migration threshold rather than a gradual response to increasing migrant influx. This threshold structure clarifies when sustained migration will drive allele replacement and reveals the presence of migration‐induced evolutionary tipping points in density‐regulated populations.

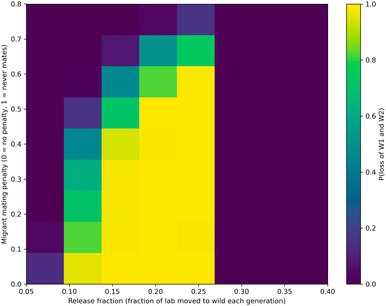

Migration‐selection balance is a foundational framework in evolutionary biology, describing how gene flow and differential fitness interact to shape allele frequencies across populations (Wright 1931; Gillespie 2004; Nielsen and Slatkin 2013). Gould and Beranek (2026) recently proposed “gene flooding” as a potential strategy for controlling invasive populations through repeated introduction of inbred individuals. Using simulation models of mosquitofish (*Gambusia*), the authors demonstrated that sustained demographic influx of inbred migrants can drive the collapse of resident alleles and potentially destabilize populations. Their study raises important questions about the evolutionary consequences of repeated asymmetric migration.

While Gould and Beranek (2026) showed that allele collapse can occur under sustained introduction of migrants, the dynamical structure governing when such collapse occurs remains unclear. In particular, it is not evident whether allele replacement under repeated demographic influx proceeds gradually as migration intensity increases or whether the system exhibits threshold behavior separating allele persistence from collapse. Clarifying this distinction is important for understanding both the evolutionary dynamics of gene flooding and the broader population genetic consequences of sustained asymmetric migration.

Classical migration‐selection theory has long documented that sustained gene flow can oppose local adaptation and shift equilibrium allele frequencies under asymmetric migration (Wright 1931; Slatkin 1973; Lenormand 2002). In many deterministic formulations, allele frequencies change continuously toward migration‐selection balance as migration increases. The present analysis asks whether repeated demographic influx in a density‐regulated population follows this gradual expectation or instead produces an abrupt threshold separating resident persistence from rapid allele collapse.

To examine this question, forward‐time simulations were conducted in Python (Van Rossum and Drake 2009) using a model closely matching the structure described by Gould and Beranek (2026). The model tracks allele dynamics at a single diploid locus under repeated introduction of migrants into a density‐regulated resident population. Resident individuals carry two alleles (W1, W2), while migrants carry two alternative alleles (L1, L2). Simulations incorporate overlapping generations, random male–female mating, Mendelian inheritance, genotype‐dependent fitness penalties, and density regulation to a carrying capacity of *K* = 1000. Individuals homozygous for the migrant‐associated deleterious allele experienced a 50% reduction in survival probability, reduced reproductive success, and fecundity reductions of 30% or 80% depending on whether one or both parents were homozygous. When populations exceeded carrying capacity, individuals were stochastically culled back to *K*.

Migration was modeled as the repeated introduction of migrants into the resident population, with a fixed migration fraction *m* assigned to each simulation run and applied in every generation across the full 100‐generation simulation. Migration fractions were varied from 0.05 to 0.4. To evaluate robustness, simulations varied migrant mating penalties and mating structure. Migrant mating penalties reduced the probability that introduced individuals entered the breeding pool, representing lower mating success of released or inbred migrants. Mating structure was varied by increasing the effective number of mating rounds, approximating multiple paternity by allowing females to mate across more than one pairing round. For each parameter combination, 50 independent stochastic replicates were performed. All simulations were implemented in Python using custom scripts. The full simulation code and analysis workflow are publicly available in GitHub to facilitate reproducibility.

Systematic variation of migration intensity reveals that resident allele loss does not occur gradually as migration increases. Instead, the system exhibits a sharp transition between resident allele persistence and collapse (Figure 1). Figure 1 illustrates the probability of simultaneous loss of both alleles across combinations of migration fraction and migration mating penalty. Each heatmap cell represents the proportion of 50 independent stochastic simulation replicates in which both resident alleles were lost by generation 100. When migrants constitute less than roughly 10%–15% of the breeding population per generation, resident alleles persist with high probability across most parameter combinations. Once migration exceeds approximately 15%–20%, collapse of both resident alleles becomes nearly deterministic. Across mating penalties and mating structures, the estimated critical migration fraction clusters narrowly around *m* ≈ 0.145 ± 0.038. These results indicate that repeated asymmetric migration generates a tipping point separating stable persistence from rapid allele replacement.

**FIGURE 1:**
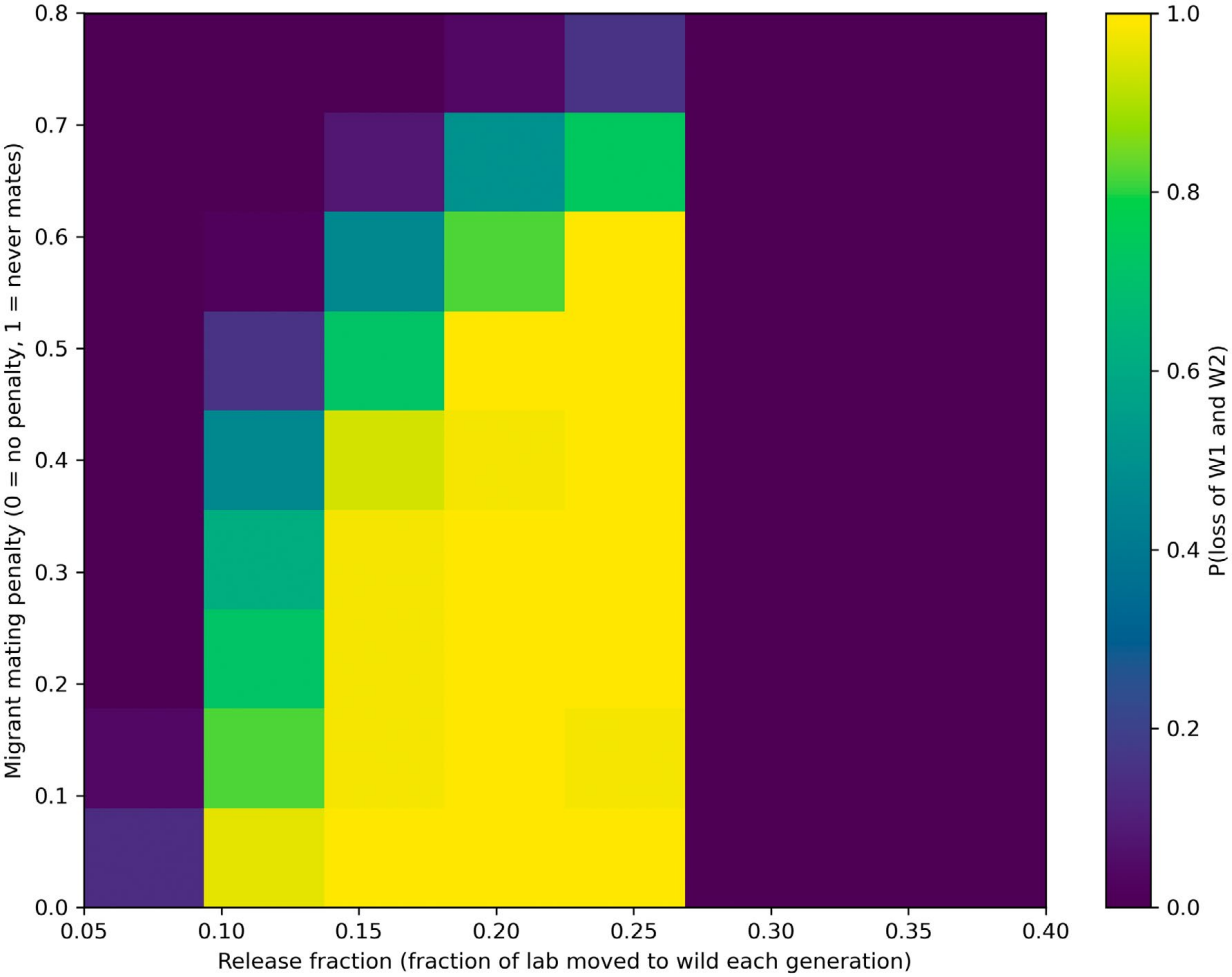
Probability of simultaneous loss of resident alleles (W1 and W2) as a function of migration fraction (m) and migrant mating penalty under baseline mating structure (multiple_paternity_factor = 1). Each cell represents the proportion of 50 independent stochastic simulation replicates in which both resident alleles were lost by generation 100. Dark regions indicate high resident allele persistence, whereas bright regions indicate near‐deterministic allele collapse. A sharp transition in collapse probability occurs between migration fractions of approximately 0.10–0.20, revealing a narrow critical threshold modulated by migrant mating asymmetry.

Importantly, this threshold behavior is robust to variation in mating topology. Increasing the opportunity for multiple paternity by allowing females to mate across additional pairing rounds had little effect on the collapse boundary, while imposing substantial mating disadvantages on migrants shifted the boundary modestly toward higher migration fractions. This suggests that demographic influx itself—rather than mating structure—is the primary control parameter governing allele replacement under sustained migration. At very high migration fractions, resident alleles can persist again under the fixed fitness‐penalty scheme applied to the migrant‐associated deleterious allele L2, producing a non‐monotonic response to increasing migration. Specifically, individuals homozygous for L2 experienced a 50% reduction in survival probability, reduced reproductive success, and fecundity reductions of 30% or 80% depending on whether one or both parents were homozygous. This high‐migration persistence likely occurs because migrant‐associated genotypes become sufficiently common at high release fractions that genotype‐dependent survival and fecundity penalties increasingly reduce their effective contribution to subsequent generations. In combination with density regulation, this allows resident alleles to persist despite continued introduction pressure. The overall system therefore exhibits nonlinear dynamics with distinct parameter regimes governing resident persistence, resident collapse, and migrant failure to establish.

These results extend the findings of Gould and Beranek (2026) by demonstrating that allele collapse under gene flooding is organized around a critical migration threshold rather than emerging as a continuous response to increasing migrant introduction. Identifying this threshold clarifies when migration‐driven replacement is expected and when resident genetic variation is likely to persist. More broadly, the analysis highlights that repeated asymmetric migration can generate tipping points in evolutionary dynamics when demographic influx interacts with density regulation and genotype‐dependent fitness. Understanding such thresholds may be important not only for evaluating gene‐flooding proposals but also for predicting the evolutionary consequences of sustained introductions in conservation translocations, population supplementation, and other contexts involving repeated demographic influx.

The results are also consistent with a broader body of theory showing that migration can swamp locally favored alleles when gene flow exceeds the counteracting force of selection, and that such swamping can be governed by critical migration rates rather than purely gradual changes in allele frequency (Barton 1992; Hu 2011; Szep et al. 2021). At the same time, the present model differs from many classical treatments because migration occurs as repeated demographic introduction into a density‐regulated population, a context in which immigration and density dependence can jointly alter adaptation dynamics (Gomulkiewicz et al. 1999; Pisa et al. 2019).

Finally, it should be noted that the present analysis evaluated migration fraction at a fixed carrying capacity; future work should examine how critical thresholds respond to variation in absolute population size and migrant number, particularly because some migration‐related dynamics may depend on absolute migrant input rather than proportional contribution alone (see Mills and Allendorf 1996).

## Author Contributions

**Andrew A. Davinack:** conceptualization (equal), formal analysis (lead), investigation (lead), validation (lead), visualization (lead), writing – original draft (lead).

## Funding

The author has nothing to report.

## Conflicts of Interest

The author declares no conflicts of interest.

## Data Availability

All python scripts, and raw data outputs are available at https://github.com/parasiteguy/migration‐threshold‐model.
